# Supplementary material for: Chromosome-Level Assemblies for the Pine Pitch Canker Pathogen Fusarium circinatum
Source: Pathogens. 2024 Jan 12;13(1):70. doi: 10.3390/pathogens13010070 (PMC10819268; doi:10.3390/pathogens13010070)
Supplement: Supplementary file 1 [file pathogens-13-00070-s001.zip › DeVos et al Figure S1.pdf]

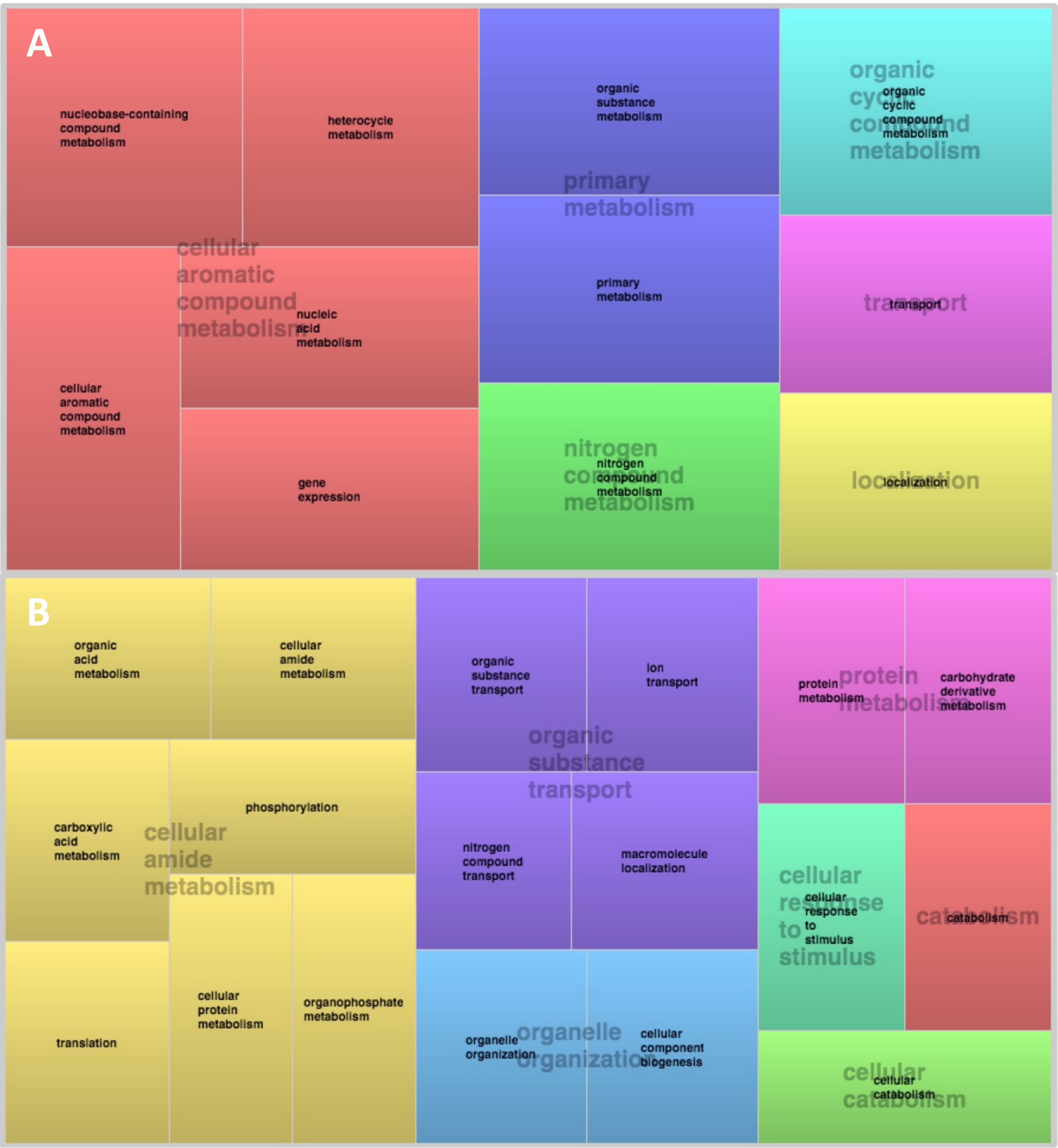

Supplemental Figure S1. REVIGO treemap summarizing GO biological process categories enriched in the accessory chromosome 12 of *F. circinatum* FSP34 (A) and KS17 (B).The enriched GO terms, together with their *P*-values, were subjected to the REVIGO web server to summarize GO terms.
