# Supplementary material for: Chromosome-Level Assemblies for the Pine Pitch Canker Pathogen Fusarium circinatum
Source: Pathogens. 2024 Jan 12;13(1):70. doi: 10.3390/pathogens13010070 (PMC10819268; doi:10.3390/pathogens13010070)
Supplement: Supplementary file 1 [file pathogens-13-00070-s001.zip › DeVos et al Figure S2.pdf]

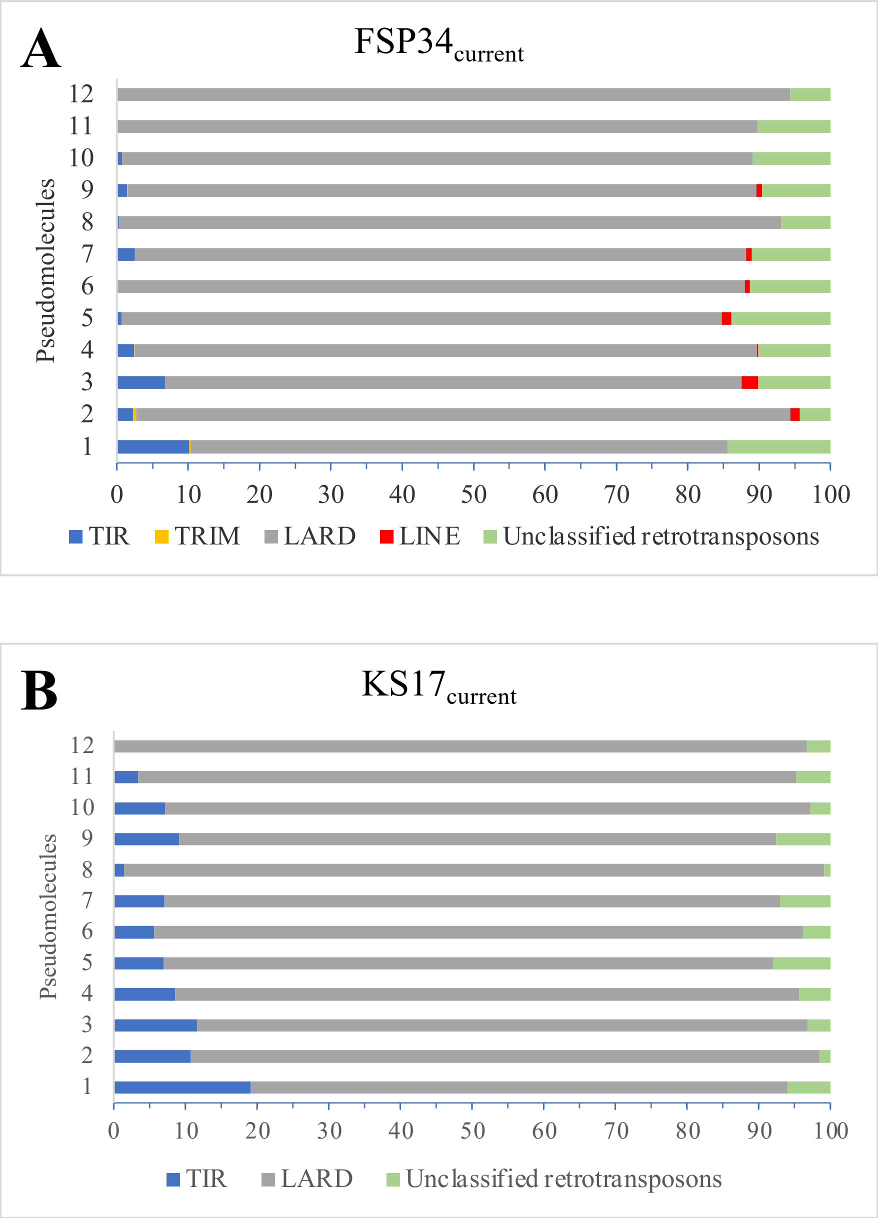

Supplemental Figure S2. Classes and orders of transposable elements (TEs) identified in $FSP34_{current}$ (A) and $KS17_{current}$ (B), based on the classification of (WICKER *et al.* 2007). The TEs are given as Class I (TRIM - Terminal Repeat transposons in Miniature, LARD - Large Retrotransposon derivatives, LINE - Long Interspersed Nuclear Elements and unclassified - non-autonomous retrotransposon), and Class II (TIR - Terminal Inverted Repeats).
