# Supplementary material for: Chromosome-Level Assemblies for the Pine Pitch Canker Pathogen Fusarium circinatum
Source: Pathogens. 2024 Jan 12;13(1):70. doi: 10.3390/pathogens13010070 (PMC10819268; doi:10.3390/pathogens13010070)
Supplement: Supplementary file 1 [file pathogens-13-00070-s001.zip › DeVos et al Figure S3.pdf]

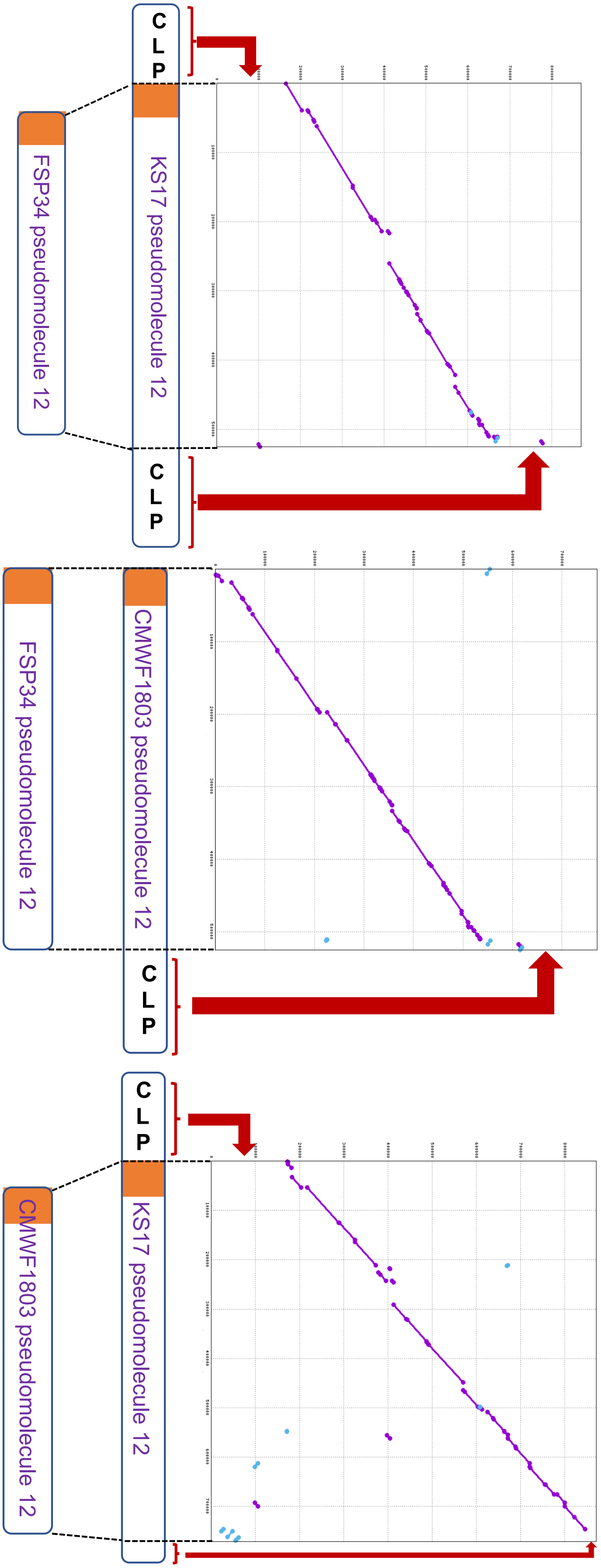

Figure S3. Sequence comparison of the chromosome length polymorphism (CLP) of chromosome 12. Genomic alignments of *F. circinatum*CMWF1803 chromosome 12 to the corresponding chromosome in FSP34 and KS17, revealed an intermediately sized chromosome 12 (771 083bp) [86]that had no distal portion (similar to FSP34). Only a partial portion of the proximal portion of KS17 that was absent in FSP34, was found. (A) Chromosome 12 comparision of *F. circinatum* FSP34 and KS17. (B) Chromosome 12 comparison of *F. circinatum* CMWF1803 and FSP34. (C) Chromosome 12 comparison of *F. circinatum* KS17 and CMWF1803. Forward matches are indicated with purple dots and reverse matches with blue dots. The orange blocks indicate the centromeric position.
