# Supplementary material for: Chromosome-Level Assemblies for the Pine Pitch Canker Pathogen Fusarium circinatum
Source: Pathogens. 2024 Jan 12;13(1):70. doi: 10.3390/pathogens13010070 (PMC10819268; doi:10.3390/pathogens13010070)
Supplement: Supplementary file 1 [file pathogens-13-00070-s001.zip › DeVos et al Figure S4.pdf]

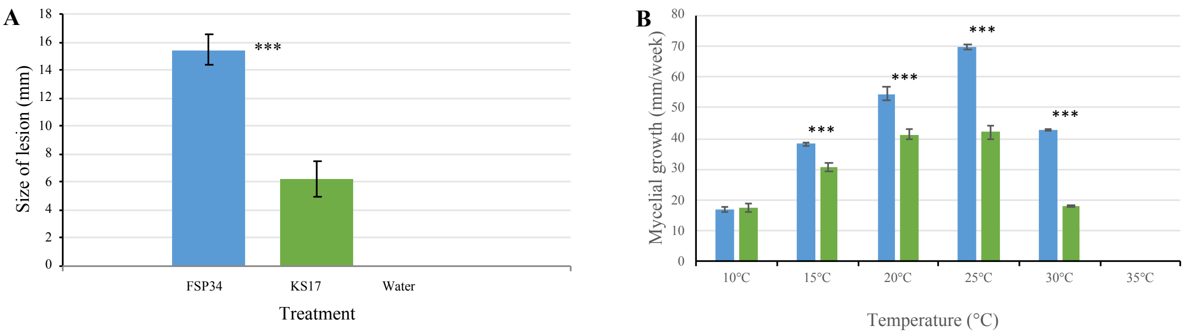


**Supplementary Figure S4.** Comparison of growth and pathogenicity of *F. circinatum* strains FSP34 and KS17. (A) Average lesion length induced by the respective strains on each of twenty *P. patula* seedlings 3 weeks after inoculation and the water control. No lesions developed on in the control treatment. (B) Average growth rate at a range of temperatures after 7 days of growth in the dark on $^{1}/_{2}$ PDA. Error bars represent the standard deviation, significant differences based on Student's t-test are indicated with an asterisk (****P* < 0.001).
